# Muscular Weakness in Individuals with HIV Associated with a Disorganization of the Cortico-Spinal Tract: A Multi-Modal MRI Investigation

**DOI:** 10.1371/journal.pone.0066810

**Published:** 2013-07-11

**Authors:** Charlotte Bernard, Bixente Dilharreguy, Michèle Allard, Hélène Amieva, Fabrice Bonnet, Frédéric Dauchy, Carinne Greib, Patrick Dehail, Gwénaëlle Catheline, for the ANRS CO3 Aquitaine cohort study group

**Affiliations:** 1 Université de Bordeaux, INCIA, UMR 5287, Talence, France; 2 CNRS, INCIA, UMR 5287, Talence, France; 3 EPHE, Bordeaux, France; 4 Université Bordeaux Segalen, INSERM U 897, ISPED, Bordeaux, France; 5 Centre Hospitalier Universitaire (CHU), Bordeaux, France; 6 Université de Bordeaux, EA 4136, Bordeaux, France; Universidad Europea de Madrid, Spain

## Abstract

Motor impairment is highly prevalent in HIV-infected patients. Here, we assess associations between peripheral muscular deficits as evaluated by the 5 sit-to-stand test (5STS) and structural integrity of the motor system at a central level. Eighty-six HIV-infected patients receiving combination antiretroviral therapy and with no major cerebral events, underwent an MRI scan and the 5STS. Out of 86 participants, forty presented a score greater than two standard deviations above mean normative scores calculated for the 5STS and were therefore considered as motor-impaired. MRI-structural cerebral parameters were compared to the unimpaired participants. Fractional Anisotropy (FA), Axial Diffusivity (AD) and Radial Diffusivity (RD), reflecting microstructural integrity, were extracted from Diffusion-Tensor MRI. Global and regional cerebral volumes or thicknesses were extracted from 3D-T1 morphological MRI. Whereas the two groups did not differ for any HIV variables, voxel-wise analysis revealed that motor-impaired participants present low FA values in various cortico-motor tracts and low AD in left cortico-spinal tract. However, they did not present reduced volumes or thicknesses of the precentral cortices compared to unimpaired participants. The absence of alterations in cortical regions holding motor-neurons might argue against neurodegenerative process as an explanation of White Matter (WM) disorganization.

## Introduction

The use of combination antiretroviral therapy (cART) has drastically improved the prognosis and the quality of life of patients infected with human immunodeficiency virus (HIV). However, neurological impairments and specifically neuromuscular problems remain highly prevalent in this population despite better control of HIV replication. These muscular impairments are also associated with daily activity limitations and participation restrictions [1], [2]. The most common motor impairments in AIDS patients are slowed movements, gait abnormality, limb incoordination, hyperreflexia, hypertonia, and muscular weakness [3], [4]. Consistent with these observation, we have shown [5] that half of adults with controlled HIV-infection had poor lower limb muscle performance as assessed through 5-Sit-To-Stand test (5STS) [6]. Given that performance on the 5STS test has been related to falls in older adults [7]–[9], HIV-1 participants infected individuals presenting poor 5STS performance should be considered as being at risk for falls.

Using MRI, several studies have demonstrated that HIV patients present White Matter (WM) alterations [10], [11]and psychomotor slowing and postural instability in HIV patients are related to these modifications [12]–[14]. In addition, finger tapping performance in this population were associated to the microstructural integrity of whole cerebrum white matter [13] as well as to the splenium of the corpus callosum [12], whereas balance scores were related to pontocerebellar tract integrity [14]. Moreover, chronic infection with HIV has been associated with skeletal muscle impairment [15]–[18], which is related to deterioration of functional ability in activities of daily living [19]. Whereas muscle weakness is well-known in the disease process, its relationship to central motor command integrity has not been investigated to date.

Taking into account these observations, we hypothesize that muscular deficits are selectively associated with central motor bundle integrity in HIV patients. In this investigation, we used Diffusion Tensor Imaging (DTI) to analyze white matter bundle microstructural organization in HIV patients presenting muscular deficits on the 5STS and compared them to unimpaired patients. These measures were evaluated through Fractional Anisotropy (FA), extracted from DTI [20], [21]. DTI is based on water motions which are differently constrained according to tissue architecture; in WM water diffusion was preferentially constrained in the direction of the bundle. This physical property is measured across FA. The more the bundle is coherent (compact and organized), the higher FA values are [22]. In order to improve pathophysiological interpretations of the FA results, the source of FA changes and its underlying tissue damage substrates are investigated using recently-described DTI parameters Axial (AD) and Radial Diffusivity (RD). In particular, AD may serve as a surrogate of axon damage whereas RD may serve as a surrogate of myelin pathology [23], [24]. In addition, global and regional cortical volumes and thicknesses will be evaluated on classical 3D-T1 to discriminate between central and peripheral effects on motor bundle integrity. All the analyses were performed first on a whole brain voxel-wise framework and were more extensively explored and confirmed using Regions Of Interest (ROIs) analysis, applied on the cortico-spinal tract and motor cortex.

## Materials and Methods

### Study set-up and design

Construction of subject groups was based on large dataset examining HIV infection, the ANRS CO3 Aquitaine cohort. This investigation constitutes an open, prospective cohort of HIV-1 infected patients [25], [26]. All adults 18 years or older who are in- or out-patients of participating hospital wards with HIV-1 infection confirmed by Western blot testing and who have provided informed consent were eligible to be enrolled in the cohort. The study was approved by the ethics committee of the local institution (CPP Bordeaux) and written informed consent was obtained for all participants. A standardized questionnaire captures different types of data: epidemiologic information (age, gender, HIV transmission category), clinical events since last medical contact (whether or not HIV-related), laboratory (plasma HIV-RNA, CD4, haemoglobin, hepatitis B and C serological status, metabolic parameters) and therapeutic treatment (cART, prophylaxis treatment, other treatment). All events are coded according to the International Classification of Diseases 10^th^ revision (ICD10).

**Figure 1:**
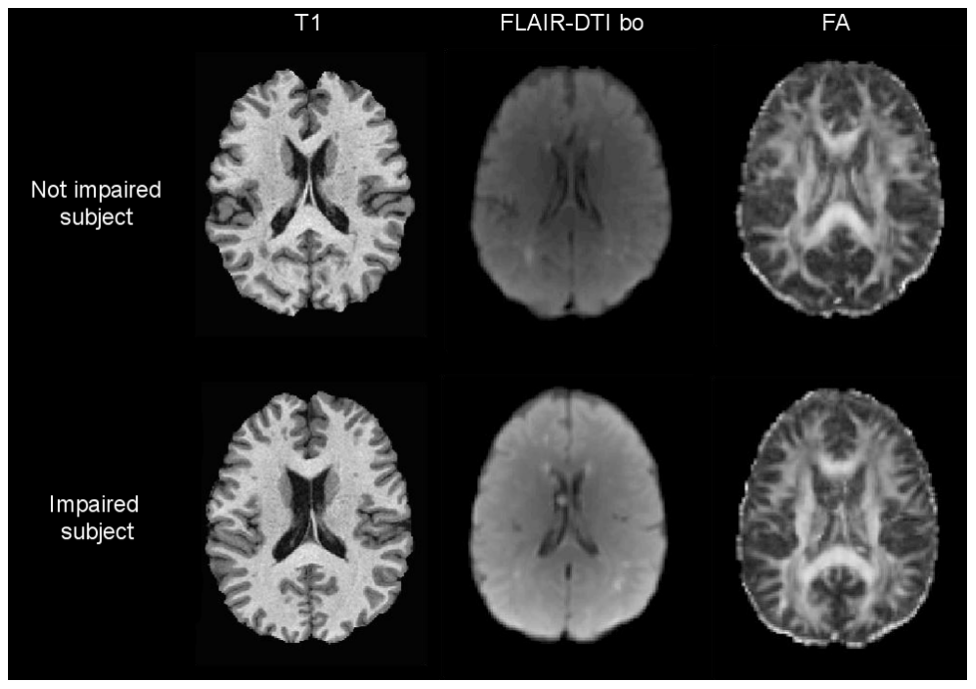
T1 scans, FLAIR DTI-b0 scans and FA maps of participants presenting sparse WM hyperintensities. These subjects are classified 1 on Fazekas scale after visual inspection. One individual example of each group was presented.

Between December 2007 and September 2009, a cross-sectional study in 324 HIV-1 infected adults from the Aquitaine Cohort was performed to assess the frequency of poor locomotor performance, including the evaluation of lower limb muscle performance with the 5STS test [6], [27], [28]. The 5STS allows for the assessment of lower limb muscle performance and is a reliable clinical tool to assess functional mobility and locomotor disability in adults [28], [29]. The 5STS test measures the time required to complete five sit-to-stand cycles at accelerated speed, recorded with a digital stopwatch. The participant sat on a standard armchair (height 45 cm) with the back against the chair and arms crossed in front of the chest. After one practice trial, the participant was instructed to rise, fully stand up and sit down again five consecutive times as fast as possible, without using the arms to push up from the chair. Timing began when the subject's buttocks leave the chair and stopped when the subject was standing up for the 5^th^ time. All subjects were able to perform the test. The test was administered by an experienced physical therapist who systematically used the same verbal procedure. For the 5STS test, poor test performance was defined by a test result of more than 2SD from the expected age-specific mean in the general population [29]. Among the 324 patients included in the locomotor study, 161 patients also had an MRI examination. Participants with dementia, history of opportunistic cerebral infection (*i.e.* toxoplasmosis), history of harmful alcohol or substance use, or absence of cART, were excluded from the present analysis (n = 46). The MRI data for each of the remaining 114 participants was inspected to discard major acquisition artifacts (n = 23), excessive head motion and technical failure during MRI post-treatment process (n = 12), or cerebral pathologies (n = 7). Using Fazekas scale [29], individuals presenting a high white matter lesion load (grade 2 and 3) were also excluded (n = 5). Finally, 86 participants were included in the present analysis, of which 40 were classified as 5STS-impaired, but none presented clinical signs of myopathy. Among the final participants, most had only small focal lesions (see Figure 1).

### MRI Acquisition

Two types of MRI scans were acquired using a 1.5 Tesla Intera system (Philips Medical Systems, Netherlands) equipped with a quadrature head coil: (1) Anatomical high resolution MRI scans of 1×1×1 mm^3^ using a 3D MPRAGE T1 weighted sequence (TR = 8.5 ms, TE = 3.9 ms, Flip angle = 8°, Matrix size = 256×224, FOV = 256×224 mm^2^ with 170 slices of 1 mm to cover the whole brain); (2) DTI scans using three repetitions of a single shot spin-echo EPI FLAIR DTI sequence (TR = 11258 ms, TE = 94 ms, Flip angle = 90°, Matrix size = 128×128, FOV = 226 mm^2^ with 150 slices of 2.5 mm, resulting in an acquisition voxel size of 1.77×1.77×2.5 mm^3^, b_value_ **= **1000s/mm^2^, six images with b_value_ = 0, 32 directions).

### DTI analysis

#### Microstructural analysis: Tract Based Spatial Statistics (TBSS)

Data processing was performed using FSL 4.1.8 software (FMRIB's Software Library; www.fmrib.ox.ac.uk/fsl/). The data were first visually checked to ensure quality of acquisition. For each subject, raw DTI images were pre-processed using Eddy Current correction and a brain mask was created using BET (Brain Extraction Tool). FA, AD and RD maps were computed by fitting a tensor model to the raw diffusion data using FDT (FMRIB's Diffusion Toolbox). TBSS was then used to perform a voxel-wise between-groups analysis of DTI metrics [30]. First, the FMRIB58 template was used as a target for all nonlinear registrations. The aligned FA maps were then averaged to create a mean FA image. Subsequently, classical TBSS processing was used (WM skeleton generation with a threshold FA value of 0.2 and projection of FA values on this skeleton). The same procedure was used to project values of AD and RD on the WM skeleton.

#### DTI indices extraction in white matter ROIs

Since whole brain voxel-based analysis derived from AD maps highlighted posterior limb of the internal capsule (PLIC), we performed a ROI analysis on this region of the Cortico-Spinal-Tract (CST) bilaterally. Binary masks of the left and right PLIC based on the JHU ICBM-DTI-81 white matter labels atlas provided in FSL were used to extract axial and radial diffusion indices. Moreover, to test the specificity of the observed effects, we extracted AD and RD in ROIs not implicated in motor control, i.e., the left and right temporal cingulum.

### Morphometric analysis

#### Macrostructural analysis: Voxel-Based Morphometry (VBM)

An optimized VBM procedure was used to analyze brain volumes [31], [32] using the VBM5 toolbox (C. Gaser; http://dbm.neuro.uni-jena.de/vbm) implemented in the Statistical Parametric Software (SPM5, Wellcome Laboratory of the Department of Cognitive Neurology, London, UK, http://www.fil.ion.ucl.ac.uk./spm/). The resulting maps were then smoothed with an isotropic Gaussian filter of 8-mm Full-Width at Half-Maximum (FWHM).

Finally, the Total Intracranial Volume (TIV) was calculated which corresponds to the sum of whole grey, whole white and whole Cerebro-Spinal Fluid (CSF) volumes.

#### Cortical thickness and volume of ROI

Cortical thickness and volumes of left and right precentral cortices (cortical regions holding central motoneurons) were obtained for each participant using Freesurfer image analysis suite (version 5.1, http://surfer.nmr.mgh.harvard.edu/); [33], [34]. Briefly, the processing includes removal of non-brain tissue [35], transformation to Talairach-like space, and segmentation of gray/white matter tissue [36], [37]. The entire cortex of each subject was then visually inspected; no subject was discarded for poor segmentation reason. After creation of the cortical representations, the cerebral cortex was parcellated into anatomical structures [38], [39].The anatomical labels were mapped to all individual brains for the quantification of average cortical thickness and average volume using automated FreeSurfer tools. In particular, cortical thickness was computed by finding the shortest distance between a given point on the estimated pial surface and the gray/white matter boundary and vice versa and averaging these two values [40].

### Statistical analyses

Comparisons between impaired and unimpaired groups were conducted for demographic, locomotor and HIV-1 related variables using Student t-tests or Mann-Whitney tests depending on variable type.

We then performed a classical voxel-based analysis to compare cerebral structures between 5STS-impaired and 5STS-unimpaired participants. Multiple linear regressions in the general linear model were performed on: (a) the FA, AD and RD maps using FSL, with the number of permutations set at 5000 and resulting statistical maps threshold set at least at p<0.05, with corrections for multiple comparisons by using the threshold-free cluster enhancement (TFCE); (b) the WM maps at a statistical threshold of p<0.05, after corrections for multiple comparisons using false discovery rate (FDR), with an extent threshold of 100 voxels in SPM5. Age, sex, laterality (and TIV when considering volumes) were entered into the models as covariates.

We next conducted statistical analyses on extracted structural parameters of different ROIs using SPSS (16.0.1, SPSS Inc.). ANCOVA was applied with age, sex and laterality as covariables. Pearson correlation analyses were also performed between 5STS scored and DTI indices. In these analyses, p-values below than 0.05 were considered as significant.

## Results

Forty participants presented low performance at the 5STS (*i.e.* score greater than 2 standard deviations above the expected age-specific mean in the general population). Mean score at the 5STS for these impaired participants was of 11.7±2.5 sec whereas for unimpaired it was of 8.1±1.5 sec (t(84) = −7.947 p<0.001) (Table 1). Impaired participants were significantly younger than unimpaired participants (46.5±1.17 years versus 50.8±1.6 years, p = 0.033), but they were not different concerning infection duration, CD4 count, viral load or CDC stage at the time of evaluation.

**Table 1:** Comparison between 5STS impaired and unimpaired subjects.

	Unimpaired 5STS subjects (n = 46)	Impaired 5STS subjects (n = 40)	p value (Student t-test or Fisher's exact test)
**age (years)**	50.8±10.8	46.5±7.4	0.033*
**men (n,%)**	44 (95%)	34 (85%)	0.092
**Right-handed (n,%)**	42 (91%)	34 (85%)	0.358
HIV-related variables			
**current CD4 (mm^3^)**	600±278	509±220	0.101
**Viral Load >50 cop/ml (n,%)**	8 (17%)	4 (10%)	0.252
**Log Viral Load**	1.82±0.67	1.68±0.25	0.187
**infection duration (years)**	12.22±6.4	14.4±5.8	0.102
**CDC stage (n,%)**			0.514
**A**	24 (53%)	21 (53%)	
**B**	13 (28%)	12 (30%)	0.514
**C**	9 (19%)	7 (17%)	
Locomotor performances			
**5STS (sec)**	8.14±1.5	11.7±2.5	0.000***
Morphometric parameters			
**global GM (cm^3^)**	645.3±78.1	657.1±71.7	0.47
**global WM (cm^3^)**	505.3±65	503±62	0.871
**left precentral volumes (cm^3^)**	6.22±0.84	6.3±0.82	0.616
**left precentral thickness (mm)**	2.75±0.14	2.77±0.16	0.609
Structural parameters			
**FA left PLIC**	0.68±0.02	0.66±0.02	0.000***
**AD left PLIC (10^−3^ mm^2^/sec)**	1.39±0.04	1.36±0.03	0.006**
**RD left PLIC (10^−3^ mm^2^/sec)**	0.58±0.02	0.58±0.01	0.933
**FA left temporal cingulum**	0.51±0.03	0.50±0.04	0.107
**AD left temporal cingulum (10^−3^ mm^2^/sec)**	3.06±0.39	3.03±0.37	0.711
**RD left temporal cingulum (10^−3^ mm^2^/sec)**	2.47±0.3	2.48±0.3	0.894

Student t-test or Fisher's exact test for demographic, HIV-related variables and locomotor performances. ANCOVA for morphometric and structural parameters with age, sex, laterality as covariables. p<0.05* ; p<0.01**; p<0,001*** (mean± SD).

### DTI analysis

**Figure 2:**
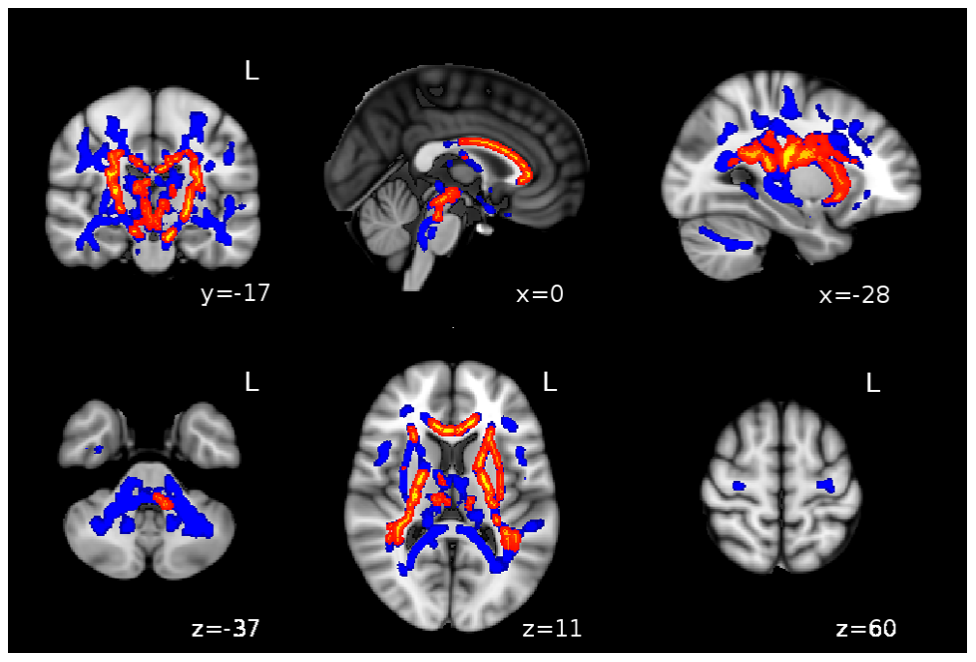
FA decrease in 5STS impaired participants. Highlighted voxels (blue or red) correspond to voxels presenting a significant reduction of FA value in 5STS impaired HIV-infected participants (n = 40) when compared to 5STS unimpaired HIV-infected participants (n = 46). Results are superimposed on MNI template with blue color corresponding to p<0.05, TFCE corrected and red to p<0.01, TFCE corrected. L = left.

**Figure 3:**
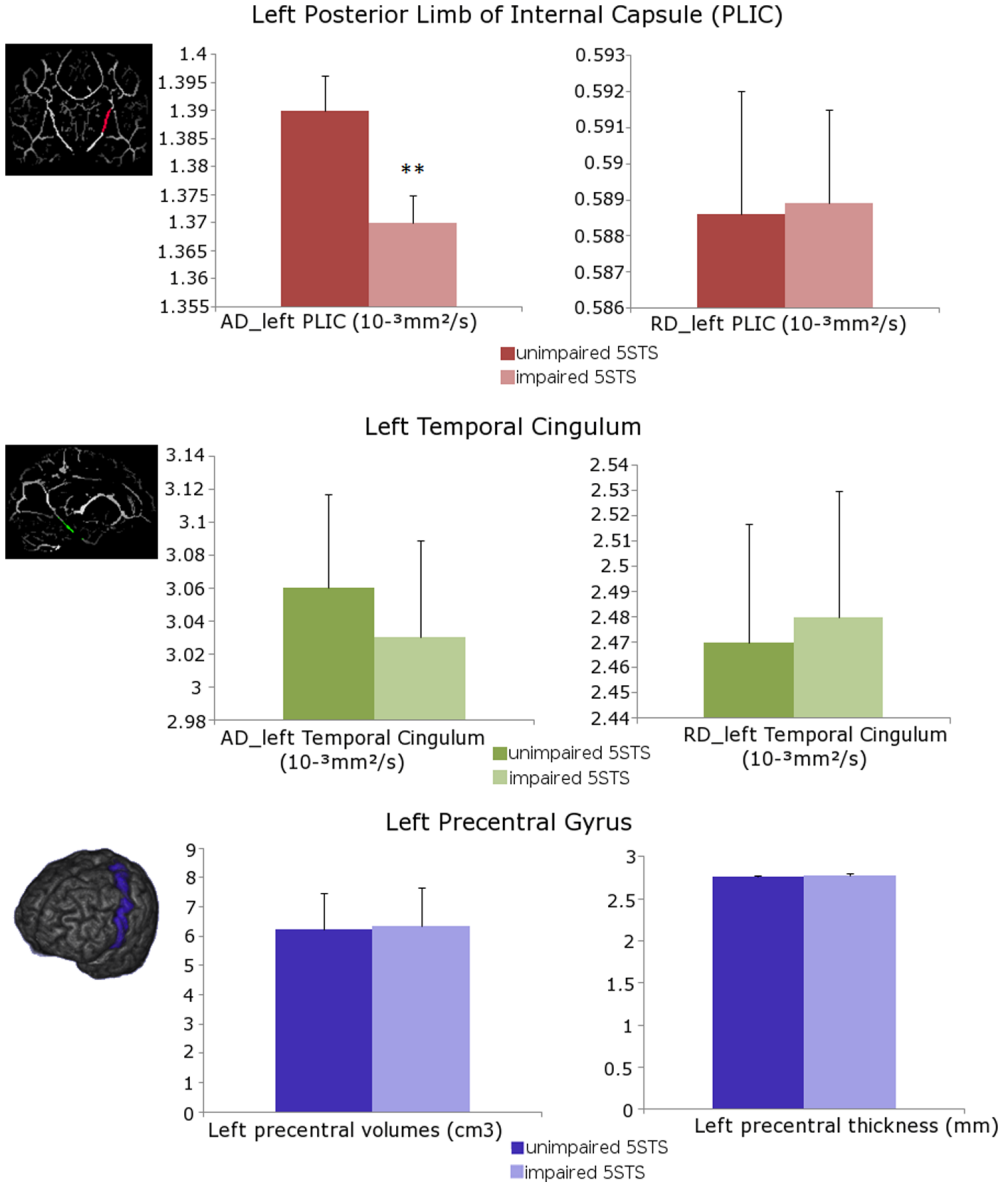
ROI comparisons between 5STS unimpaired (n = 46) and impaired (n = 40) participants. AD and RD of left PLIC (upper raw) and left temporal cingulum (middle raw) and volume and thickness of left precentral region (bottom raw) were compared (ANCOVA with age, sex and laterality as covariables, ** p<0.01).

**Figure 4:**
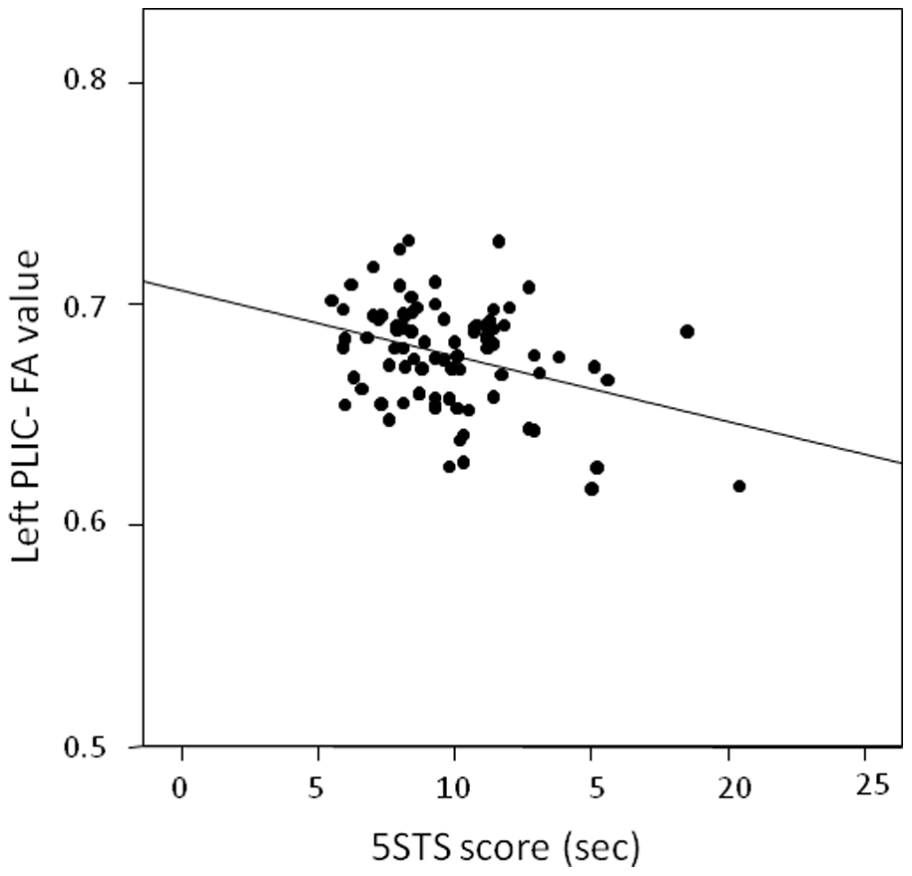
Scatter plot showing correlation between left PLIC-FA values and 5STS score. The analysis includes all participants.

The voxel-wise analysis performed on FA maps demonstrated that 5STS impaired participants had lower FA values at the level of various tracts and especially at the level of motor tracts, *i.e.* the cortico-spinal tracts (CST), the middle cerebellar peduncles, the medial lemniscus and the corpus callosum (CC) (all differences p<0.05, TFCE corrected, Figure 2). It is noteworthy, that impaired participants presented a reduced FA all along the CST, in the midbrain, in the internal capsule and also in the proximal part of CST (the corona radiata close to precentral gyri, which underlies the specificity of the observed relationship). When a more stringent threshold (p<0.01, TFCE corrected) was applied, lower FA values persisted in the CST and in the anterior CC (Figure 2). Impaired participants also presented a smaller value of AD at the proximal level of the left CST (p<0.05 TFCE corrected), whereas no difference was observed for RD. Subsequent ROI analyses confirmed that impaired participants presented significant lower AD values at the level of the left posterior limb of the internal capsule (PLIC) (1.36±0.03 10^−3^ mm^2^/sec) when compared to unimpaired participants (1.39±0.04 10^−3^ mm^2^/sec, F(4,85) = 8.038, p = 0.006). No difference was observed for RD values at this level between impaired and unimpaired participants (0.58±0.01 10^−3^ mm^2^/sec *versus* 0.58±0.02 10^−3^ mm^2^/sec respectively for, F(4,85) = 0.38, p = 0.539) (Figure 3). The same results were obtained for the right hemisphere (data not shown). In order to appreciate regional specificity of this difference, we explored the group differences for AD and RD values along the left temporal cingulum, which is not involved in motor control. At the level of this white matter bundle, no difference was detected between unimpaired and impaired participants (3.06±0.39 10^−3^ mm^2^/sec *versus* 3.03±0.37 10^−3^ mm^2^/sec respectively, for AD values, F(4,85) = 0.222, p = 0.639, and 2.47±0.3 10^−3^ mm^2^/sec *versus* 2.48±0.3 10^−3^ mm^2^/sec respectively for RD values, F(4,85) = 0.918, p = 0.341). A significant correlation was observed between 5STS score and left PLIC- FA values (*r* = −0.337, p<0.001, Figure 4) as well with left PLIC-AD values (*r* = −0.197, p<0.034).

### Morphometric analysis

When global grey or white matter volumes were compared between the two groups, no significant difference was observed. Similarly, no regional differences were observed using VBM analysis comparing the 2 groups.

No difference emerged for comparisons between unimpaired and impaired participants regarding volume (6.22±0.84 cm^3^ *versus* 6.30 ±0.82 mm^3^ respectively, F(4,85) = 0.007, p = 0.932) or cortical thicknesses (2.75±0.14 mm^3^ *versus* 2.77±0.16 respectively, F(4,85) = 0.032, p = 0.858) in left precentral regions (Figure 3). In the same way, no difference was observed when right precentral regions were examined (data not shown).

## Discussion

The present study shows that HIV-1 participants presenting motor deficits exhibited a selective decrease in the coherence of central motor tracts in the CST, compared to participants without deficits. In contrast, no macrostructural differences were revealed neither in volume nor in thickness of cortical precentral regions (regions holding cellular bodies of motoneurons projecting in CST) between the two groups. Neither age, nor HIV-related clinical variables are related to WM abnormalities. DTI allows examination of WM abnormalities of the motor tracts in these impaired 5STS subjects, in normal appearing WM on conventional MRI. Since no abnormalities in motor grey matter were observed, Wallerian degeneration cannot explain these WM abnormalities.

Voxel-wise analysis, allowing no *a priori* hypothesis on cerebral regions involved, revealed that participants with impaired motor performance also present low FA values bilaterally all along the CST (from its brainstem to its cranial portions). In animal studies [41]–[43] as in human studies [44]–[46], quantitative FA measures are believed to reflect axonal density and/or myelin content. No such difference was observed in non-motor tracts (*i.e.* temporal cingulum), this finding supports the specificity of the relationship between peripheral motor deficit and central motor bundle damage. Thus, we could posit that HIV-1 participants with motor deficits also present axonal degeneration and/or dysmyelination of the CST. This finding is in accordance with a number of studies demonstrating a correlation between morphological alterations of the CST and motor disturbances. Ischemic stroke occurring at the level of the CST was associated with Wallerian degeneration of this tract, as revealed through FA measure [47]–[49]. In Multiple Sclerosis (MS), a disease characterized by focal WM inflammatory lesions, FA of the CST was related to pyramidal functional scores [50] and muscular weakness [51]. Finally, in Amyotrophic Lateral Sclerosis (ALS), a neurodegenerative process which takes place at the level of motor neuron cell bodies and spreads to WM efferents [52]–[57], symptom severity is associated with a decrease of FA of CST [55], [58]–[64]. In all these studies, CST damage is related to a top-down neurodegenerative process occurring either after a direct lesion of the central nervous system (stroke or MS) or after motor neuron impairment (ALS). Nevertheless, the assumption of a top-down driven neurodegenerative process is unlikely in our study because locomotor impaired HIV patients with cerebral pathology were excluded from our analysis. In support of this possibility, motor cortex integrity was assumed since neither volume nor thickness decreases were observed in cortical motor areas. Otherwise, the presence of white matter damage specifically in the motor tracts (and nowhere else), and the fact that both HIV-1 groups present comparable viral loads, CD4-count, infection duration and CDC-clinical stage argue against non specific toxic effect of HIV-infection on CST microstructure integrity. This conclusion is also supported by previous observations indicating that neither history of polyneuropathy nor cART treatment are related to 5STS impairment [5]. In sum, these data support the hypothesis that the FA decrease observed in the CST of motor-impaired HIV patients is a central adaptation (mal-adaptative plasticity?) to peripheral impairment, i.e. muscular weakness frequently observed in HIV patients. We could posit that a decrease of muscular exercise due to muscular weakness could induce an adaptation of the supraspinal motor command. Even if the cross-sectional design of our study does not give us access to the dynamic of the FA decrease during the course of the disease, we can speculate that a vicious circle could set in, mutually worsening peripheral muscle weakness and motor central bundle damage. However, we cannot exclude a functional adaptation of neurons of the motor cortex (not evaluated here) associated with a decrease in motor ability [65], which might affect WM efferents and induce a decrease of CST coherence. Also, we cannot exclude the possibility that CST compromise occurs earlier than grey matter atrophy or even that it is an independent phenomenon. Accordingly, a recent brain MRI study performed on myotonic dystrophies revealed large white matter changes which exceed those for grey matter. This observation led the authors to conclude against Wallerian degeneration as the major cause of white matter impairment in this muscular pathology [66].

At this stage, the question arises as to the pathophysiological mechanism underlying white matter tract injury. Whole brain analyses contrasting 5STS unimpaired and impaired motor participants revealed that specific FA decreases observed in 5STS-impaired participants also demonstrate AD decreases at the same level (the left PLIC), while RD remains unchanged in these same individuals. These results were confirmed and extended to right PLIC by ROIs analyses. The FA decrease seems to be driven by a relative AD decrease. According to rodent studies, RD is a fairly consistent biomarker of myelination rates [24], [67]–[72] whereas AD rather reflects axonal changes [41], [42], [67]. The concomitant decrease of FA and AD could then be driven by a decreased fiber tract organization (caliber change, decreased density and packing of the axons). Even if biological interpretations of the various DTI indices still remain a major concern in the MRI literature [73], [74], our results remain consistent with a bottom-up effect.

### Methodological considerations

Recently, it has been demonstrated that diffusivity metrics are greatly contaminated by voxels containing CSF due to WM atrophy [75]–[77]. Our results do not appear to be susceptible to this misinterpretation since locomotor-impaired participants did not present lower WM volumes compared to unimpaired participants. WM hyperintensities have also been shown to impact DTI indices measures [75]. Here again, this interpretation could be discounted since participants included in the study generally presented normal appearing WM.

In conclusion, our results suggest that motor impaired HIV-1 participants present central motor tract disorganization which could be an adaptation to their peripheral impairment rather than a neurodegenerative process. This maladaptative process could support increased damage in white matter bundles throughout the course of the disease and then exacerbate the initial muscular weakness, increasing the risk of falls and disability in these individuals [78], [79]. However, given the recent studies on cerebral plasticity following physical training, muscle training in HIV-1 participants may be able to counteract this maladaptive process and may facilitate rehabilitation.
